# Characteristics of breathing‐adapted gating using surface guidance for use in particle therapy: A phantom‐based end‐to‐end test from CT simulation to dose delivery

**DOI:** 10.1002/acm2.14249

**Published:** 2023-12-21

**Authors:** Abdallah Qubala, Jehad Shafee, Thomas Tessonnier, Julian Horn, Marcus Winter, Jakob Naumann, Oliver Jäkel

**Affiliations:** ^1^ Heidelberg Ion Beam Therapy Center (HIT) Heidelberg Germany; ^2^ Faculty of Medicine University of Heidelberg Heidelberg Germany; ^3^ National Center for Radiation Research in Oncology (NCRO) Heidelberg Institute of Radiation Oncology (HIRO) Heidelberg Germany; ^4^ Saarland University of Applied Sciences Saarbruecken Germany; ^5^ Department of Medical Physics in Radiation Oncology German Cancer Research Center (DKFZ) Heidelberg Germany; ^6^ National Center for Tumor Diseases (NCT) Heidelberg Germany

**Keywords:** breathing‐adapted gating, commissioning, end‐to‐end testing, ion beam therapy, particle therapy, surface‐guided radiotherapy

## Abstract

To account for intra‐fractional tumor motion during dose delivery in radiotherapy, various treatment strategies are clinically implemented such as breathing‐adapted gating and irradiating the tumor during specific breathing phases. In this work, we present a comprehensive phantom‐based end‐to‐end test of breathing‐adapted gating utilizing surface guidance for use in particle therapy. A commercial dynamic thorax phantom was used to reproduce regular and irregular breathing patterns recorded by the GateRT respiratory monitoring system. The amplitudes and periods of recorded breathing patterns were analysed and compared to planned patterns (ground‐truth). In addition, the mean absolute deviations (MAD) and Pearson correlation coefficients (PCC) between the measurements and ground‐truth were assessed. Measurements of gated and non‐gated irradiations were also analysed with respect to dosimetry and geometry, and compared to treatment planning system (TPS). Further, the latency time of beam on/off was evaluated. Compared to the ground‐truth, measurements performed with GateRT showed amplitude differences between 0.03 ± 0.02 mm and 0.26 ± 0.03 mm for regular and irregular breathing patterns, whilst periods of both breathing patterns ranged with a standard deviation between 10 and 190 ms. Furthermore, the GateRT software precisely acquired breathing patterns with a maximum MAD of 0.30 ± 0.23 mm. The PCC constantly ranged between 0.998 and 1.000. Comparisons between TPS and measured dose profiles indicated absolute mean dose deviations within institutional tolerances of ±5%. Geometrical beam characteristics also varied within our institutional tolerances of 1.5 mm. The overall time delays were <60 ms and thus within both recommended tolerances published by ESTRO and AAPM of 200 and 100 ms, respectively. In this study, a non‐invasive optical surface‐guided workflow including image acquisition, treatment planning, patient positioning and gated irradiation at an ion‐beam gantry was investigated, and shown to be clinically viable. Based on phantom measurements, our results show a clinically‐appropriate spatial, temporal, and dosimetric accuracy when using surface guidance in the clinical setting, and the results comply with international and institutional guidelines and tolerances.

## 1 INTRODUCTION

Tumor motion management throughout the entire radiotherapy (RT) workflow, including image acquisition, treatment planning, patient positioning and irradiation, poses a major challenge in delivering the prescribed dose to mobile tumor targets in sites such as thorax and abdomen.^1^, ^2^, ^3^, ^4^, ^5^, ^6^ The process of motion management is aimed to decrease the dose to organs at risk (OARs), and to accurately cover the clinical target volume (CTV).^7^, ^8^, ^9^ For instance, respiratory‐induced tumor motion demonstrates one of the intra‐fractional patient anatomy variations in abdominal treatment sites (such as pancreas and liver). These variations may cause image artifacts, distort the assessment of the tumor trajectory and lead to an undesired dose distribution to the target volume and OARs (such as motion interplay effect).^7^, ^10^, ^11^, ^12^, ^13^ These challenges become more essential in implementing tumor treatment approaches that use high doses with extremely steep dose gradients.^14^, ^15^, ^16^, ^17^ Compared to photons, ion beams exhibit this latter advantage by delivering a higher conformal dose distribution to a deeply located target with a less integral dose to OARs.^14^, ^18^, ^19^, ^20^

Nowadays, various treatment approaches are employed in clinical practice to address intra‐fractional tumor motion during both treatment planning and dose delivery. These strategies include (i) expanding the internal margin of the target volume using the internal target volume (ITV) concept,^21^, ^22^ (ii) utilizing abdominal compression to restrict the tumor motion (e.g., in liver treatments),^23^ (iii) employing breath‐hold methods,^23^, ^24^ (iv) implementing tumor tracking,^24^, ^25^ and (v) using breathing‐adapted gating approaches to attempt tracking the patient breathing, and irradiating the tumor target during specific breathing phases.^26^, ^27^, ^28^, ^29^, ^30^, ^31^, ^32^ For the latter, four‐dimensional computed tomography (4DCT) is used to reconstruct images at specific breathing phases based on either amplitude or phase.^28^, ^33^, ^34^, ^35^, ^36^ Different respiratory monitoring systems (RMSs) are used for both 4DCT imaging and gating, including pressure sensors, skin‐surface camera systems, radiofrequency‐based systems and fiducial markers in combination with image‐guided RT.^23^, ^37^, ^38^, ^39^, ^40^, ^41^

At Heidelberg Ion Beam Therapy Center (HIT), three optical surface‐guided RT (SGRT)^38^ systems, the AlignRT, SimRT, and GateRT (VisionRT Ltd, London, United Kingdom) have been installed between 2019 and 2021: (i) AlignRT and GateRT at the ion beam gantry treatment room, and (ii) SimRT at a CT scanner in the radiation oncology department. SimRT is used for 4DCT‐based treatment planning.

AlignRT is implemented for patient positioning and tracking during the RT course without any additional dose.^38^, ^42^, ^43^ GateRT is applied as a respiratory gating method which records the real‐time respiratory deviation using the patient skin surface as an external surrogate, and assumes that the tumor position is a function of the external surrogate motion.^8^ For these three applications of SGRT, the current and reference patient skin surface are compared within a user‐defined region of interest or patch.

In this work, we present a comprehensive phantom‐based end‐to‐end (E2E) testing of a respiratory‐adapted gating method based on surface guidance at an ion beam gantry. The experiments were based on international guidelines.^23^, ^39^, ^40^, ^44^, ^45^, ^46^ First, we investigated the accuracies and dependencies of all SGRT systems used in this work. Second, we studied the reliability, reproducibility, temporal and spatial accuracy of GateRT. Then we evaluated the dosimetric and geometric properties using (i) an ionization chamber and (ii) radiochromic films. Finally, we tested the implementation of a treatment planning system (TPS)‐based tool to attempt detecting the camera system occlusions during the treatment planning process. Such camera occlusions can distort the field of view (FOV) of the patient skin surface during the gated irradiation, especially when using different gantry and couch angles. Limitations of the SGRT systems will be also reported.

## 2 METHODS AND MATERIALS

### 2.1 Ion beam gantry treatment room and CT scanner

**FIGURE 1:**
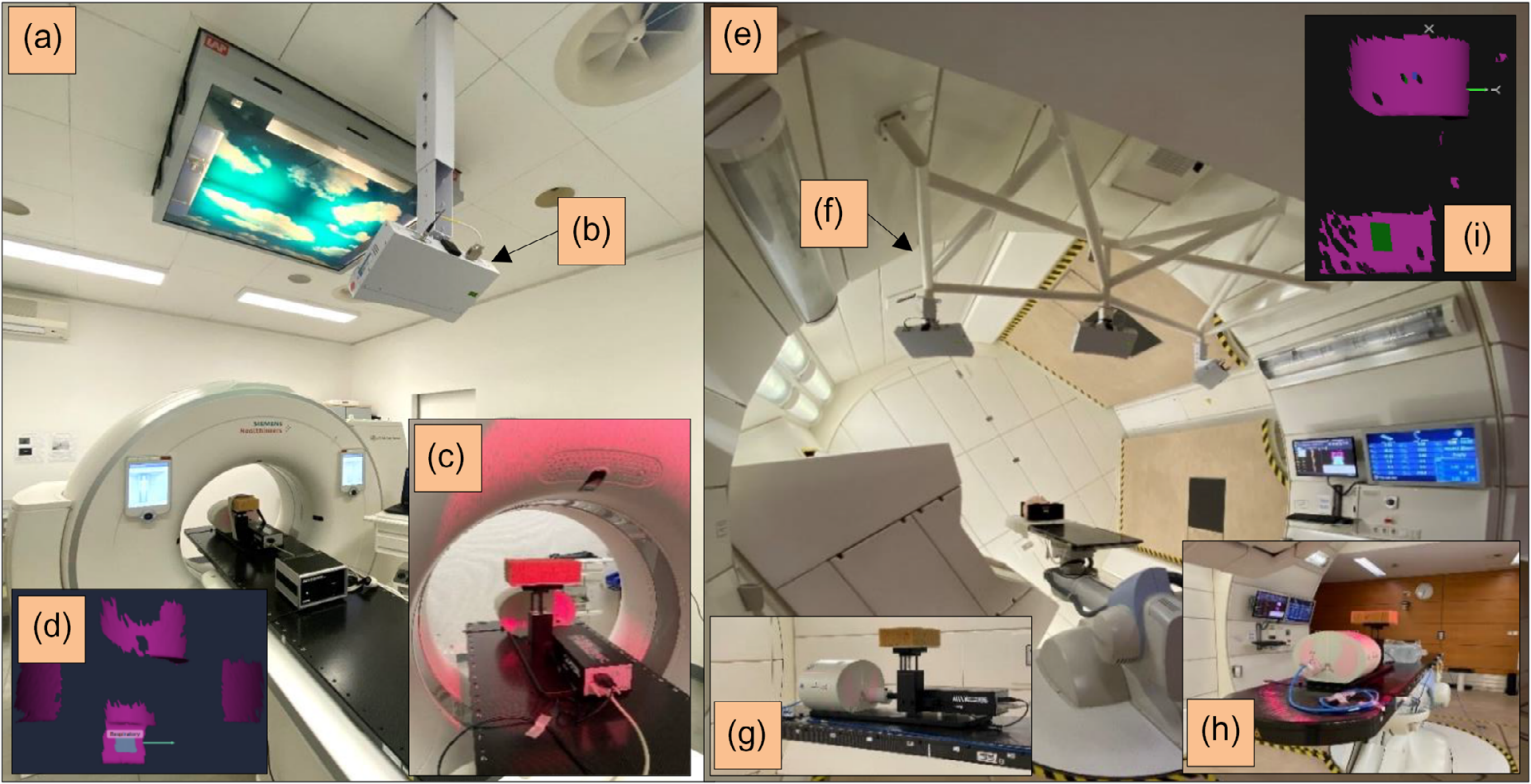
Left: CT scanner room (a) including the SGRT system (b), the experimental setup using the CIRS phantom (c), and the reference capture used for the 4DCT reconstruction including the patch in grey (d). Right: Gantry treatment room with the beam nozzle at 240° at HIT (e) including the SGRT systems mounted on the installation (f), the experimental setup containing the CIRS phantom and ionization chamber (g), (h), and the reference capture performed for the gating irradiation with the patch in green (i). In both setups, a brown sponge as a surrogate was used. 4DCT, four‐dimensional computed tomography; CIRS, computerized imaging reference systems; SGRT, surface‐guided RT.

The experiments of AlignRT and GateRT in this study were performed at the ion beam gantry treatment room at HIT, which features an isocentric gantry with a length of 25 m, a diameter of 13 m, and the ability to rotate around 360° (Figure 1e). Despite the considerable mass of the gantry (670 tons), an accuracy of less than 1 mm in beam position is achieved. HIT is a synchrotron‐based facility delivering protons, and carbon and helium ions for clinical use with raster‐scanning techniques.^19^, ^47^, ^48^, ^49^

Planar kV imaging is used for patient position verification and final alignment. The CT scans for the treatment planning were performed on a SOMATOM Confidence CT scanner (Siemens Healthineers, Erlangen, Germany) under the same respiratory conditions as during irradiation (Figure 1a).

### 2.2 Optical SGRT systems

The camera modules of the SGRT systems (AlignRT, SimRT, and GateRT) used in this work consist of two image sensors and a projector that displays an optical random speckle patterns on the patient skin surface. The only difference between the three modules installed in our institution is the number of camera systems used for each module (Figure 1a,e).

SimRT consists of a single camera pod placed on the ceiling above the foot of the CT couch with a view into the CT‐bore, and can be connected with the CT scanner to receive the beam on/off status (Figure 1a). A time‐resolved 4DCT for respiratory‐adapted gating can be generated by resorting the CT projections into the different breathing phases by using the recorded respiratory pattern detected from the patient skin surface. So, several 3DCT images can be generated from the 4DCT dataset. SimRT has been already described in detail.^50^ AlignRT which has been already described in detail, consists of three camera systems mounted to the bearing of our ion beam gantry^51^ (Figure 1e), and two of them are used for GateRT which is able to track the spatial respiratory variation of a user‐defined patch on a selected position of the patient skin surface in millimetre during treatment (Figure 1i).

Depending on the gating method used, (phase‐ or amplitude‐based), GateRT automatically turns on/off the radiation when the respiratory pattern moves inside/outside the user‐defined gating window.^38^ As GateRT does not directly measure the breathing phase, the real‐time breathing curve is initially assessed based on its amplitude. Following a learning period to establish signal stability, GateRT subsequently divided the breathing signal into distinct phases. These phases are then utilized to configure the gating window.

### 2.3 Respiratory motion phantom and characteristics of simulated respiratory curves

**FIGURE 2:**
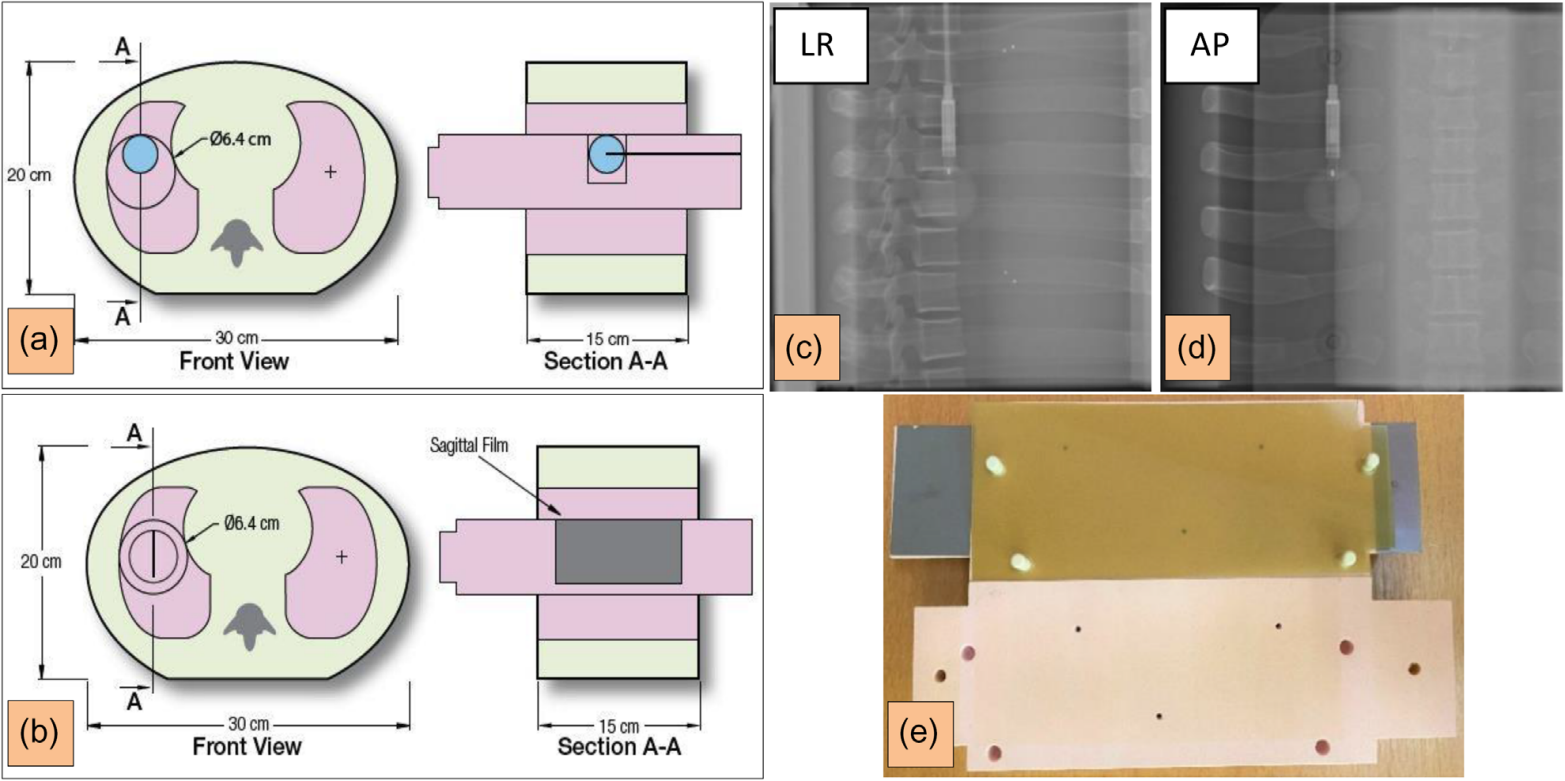
Left: Cross‐section images of the CIRS Dynamic Thorax phantom (a)–(b) including inserts for both PinPoint chamber 0.03 cm^3^ (a) and radiographic EBT3 films (b) used in this work. (Figure courtesy Sun Nuclear GmbH)^53^ Right: Planar kV images (c)–(d) in LR and AP illustrating the PinPoint chamber inside the lung, and an EBT3 film positioned inside the insert (e). A motion uncertainty of 0.1 mm can be achieved. AP, anteroposterior; CIRS, computerized imaging reference systems; LR, left‐right.

The CIRS Dynamic Thorax phantom 008A (Computerized Imaging Reference Systems, CIRS, Norfolk, VA) was used to simulate prespecified respiratory patterns for both the lung in anteroposterior direction (AP), left‐right direction (LR), inferior‐superior (IS) direction, and the surrogate platform in AP (Figure 2). The phantom consists of anthropomorphic tissues as tissue‐equivalent lung, soft tissue, cortical and trabecular bones.

In addition, a brown sponge of 10 × 10 × 20 cm^3^ (Figure 1) was used and fixed on the rigid phantom platform which can be detected by the SGRT systems (SimRT and GateRT). The phantom has been already validated and described in detail.^50^ Planned regular and irregular respiratory patterns with different periods and peak‐to‐peak amplitudes were applied to the ground‐truth by the CIRS phantom (Table 1).

**TABLE 1:** Characteristics of used respiratory patterns.

Respiratory pattern	T ± SD [s]	A ± SD [mm]
**Cos*^4^*** **C1‐6z**	2	2
	4	2
	2	8
	4	8
	2	16
	4	16
**Volunteer with a regular breathing pattern** **RV1‐4**	7.43 ± 0.53	10.18 ± 1.13
	6.75 ± 0.16	16.43 ± 0.97
	7.74 ± 0.53	18.02 ± 2.12
	6.31 ± 0.20	20.87 ± 1.59
**Volunteer with an irregular breathing pattern** **IRV1‐4**	9.39 ± 3.68	15.77 ± 2.70
	5.85 ± 1.63	14.15 ± 3.75
	5.42 ± 1.07	14.42 ± 3.36
	5.09 ± 2.33	14.27 ± 6.68

Abbreviations: A, peak‐to‐peak amplitude; SD, standard deviation; T, period.

The cos^4^ patterns were simulated in MATLAB 2022a (MathWorks, Natick, MA) and given according to the following formula:

(1) $$x\left(t\right)=A\cdot \cos{\left(\pi \cdot \frac{1}{T}\cdot t\right)}^{4}$$

where x(t) is the respiratory pattern at the given time t, A is the peak‐to‐peak amplitude and $\frac{1}{T}$ the cosine wave frequency. All respiratory patterns were tracked for at least 60 s. Respiratory patterns of volunteers were acquired using the Anzai system and then transferred to the CIRS phantom. The Anzai system has been already described in detail.^50^

### 2.4 Preparation for SGRT‐based gated irradiation

Two kinds of CT scans were required for our dosimetric experiments using the CIRS phantom: (i) planning 3DCT at 0% inhale for treatment planning, and (ii) 4DCT using SimRT for assessing the tumor trajectory. The 4DCT was divided into 10% phases of inhale and exhale. Our Siemens CT scanner classifies the breathing curve into inhale (0%–100%) and exhale (0%–100%) phases, whereas GateRT divides the same curve, setting the maximum for 100% inhale and the minimum for 50% exhale. This results in a proportional relationship, where 20% inhale or exhale in the CT curve corresponds to 10% inhale or exhale in the GateRT curve (Figure S1).

The cos^4^ pattern was used in all dosimetric measurements. For absolute dose measurements using the ion chamber, a peak‐to‐peak amplitude of 16 mm for the surrogate, 10, 10, and 16 mm for the tumor targets inside the lung (AP, LR, and IS), respectively, were chosen. For relative measurements, a peak‐to‐peak amplitude of 20 mm for both the surrogate (AP) and radiochromic film insert inside the lung (IS), respectively, was used. A period of 4 s was selected for both measurements. The CT parameters used during image acquisition are presented in Table 2.

**TABLE 2:** CT parameters on the Somatom Confidence CT scanner used for this work.

CT Parameter	3DCT	4DCT
**Voltage [kV]**	120	120
**Effective current [mAs]**	300	50
**Slice collimation [mm]**	1.2 × 16	1.2 × 16
**Slice thickness [mm]**	3	3
**Rotation time [s]**	1	0.5
**Pitch**	0.85	0.09
**Reconstruction kernel**	Br40	Br38
**Field of view, FOV [mm]**	500	500

**FIGURE 3:**
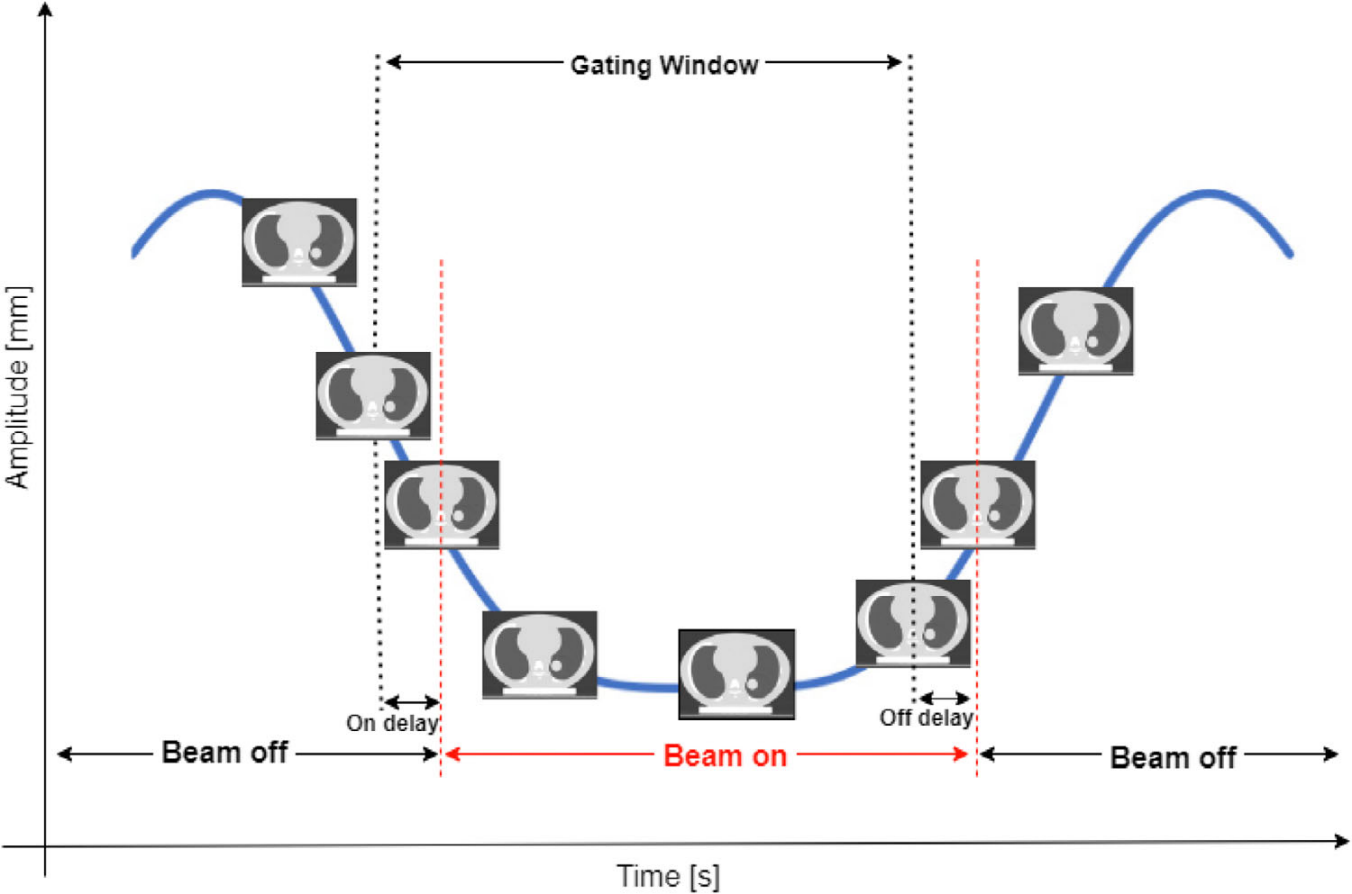
Schematic illustration of the gated irradiation with beam on/off delays. Beam is on when target is within the gating window. Otherwise, beam is off.

The treatment plans using carbon ions were generated by RayStation TPS (RS11B; RaySearch Laboratories, Stockholm, Sweden), and the gating window for GateRT was defined by assessing the 4DCT datasets in RayStation TPS. In this work, the gating window was chosen between 30% exhale to 30% inhale which means a gating window of minimal 15% to maximal 65% in GateRT (Figure S1 and Figure 3). The treatment planning is described in Section 1.

To position the CIRS phantom in the treatment room by AlignRT prior to the position verification with planar kV imaging, the reference surface was also created in the TPS and exported to AlingRT including the information of the irradiation plan (RTPlan and RTStruct). A detailed description of how patient positioning using SGRT works is included in our previous study.^51^

### 2.5 SGRT system accuracy

The absolute positioning and tracking accuracy of AlignRT at the ion beam gantry have been investigated in our previous study, including phantom measurements and patient measurements of different body sites.^51^ Furthermore, the spatial, temporal, and reconstruction accuracy of SimRT at the Siemens CT scanner have recently been described in our previous work.^50^ In this work, the spatial, temporal, and dosimetric accuracy of GateRT will be presented.

### 2.6 Gantry and couch angle dependency

**FIGURE 4:**
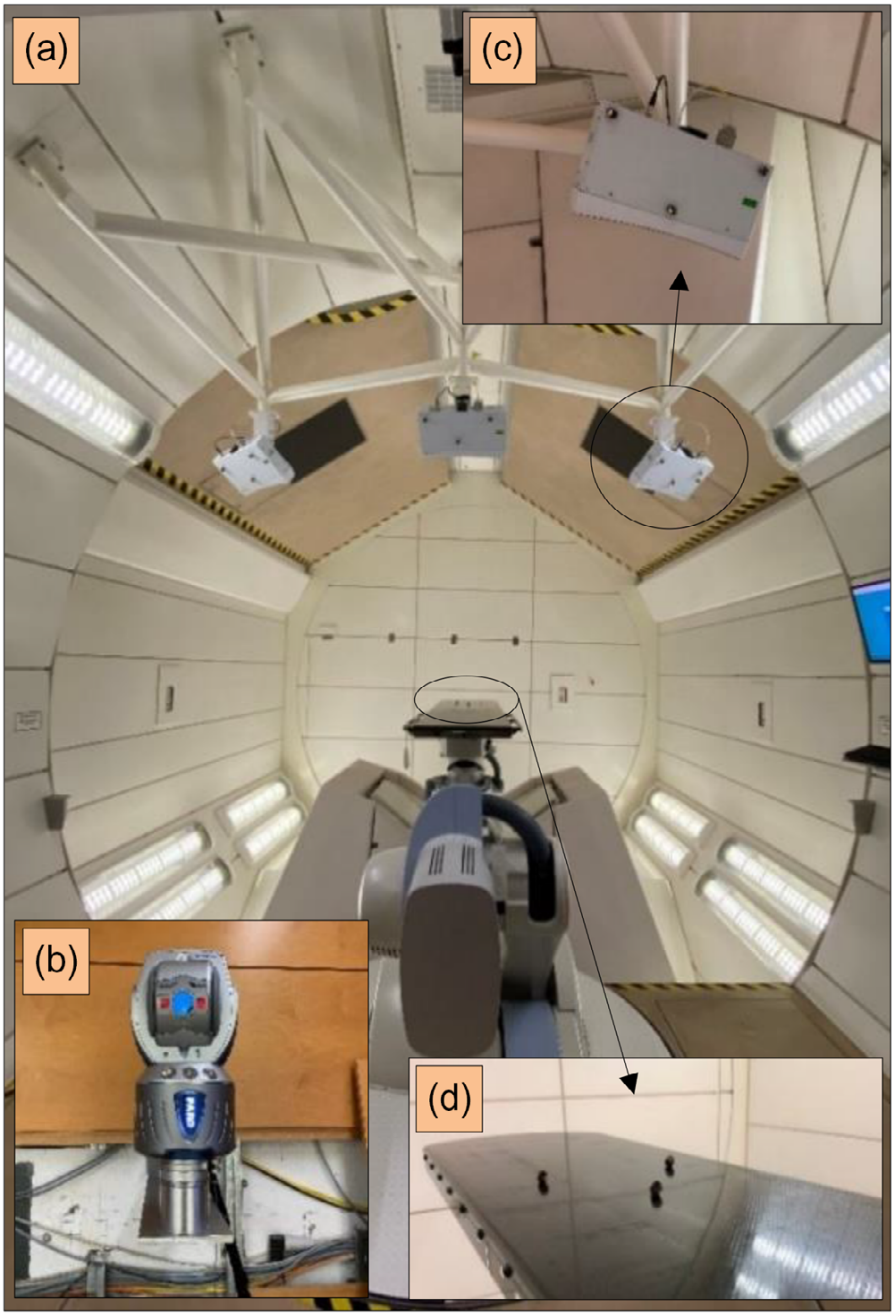
The experimental setup using the FARO 3D laser tracker with the beam nozzle at 180° at the gantry treatment room of HIT (a) including the laser tracker placed on the fixed north wall (b), an SGRT system mounted on the celling bracket (c), and the treatment couch positioned at the room isocentre (d). The black markers illustrated in the Figure are the laser tracker markers (c), (d). HIT, Heidelberg Ion Beam Therapy Center.

The gantry angle dependency was investigated to verify any transfer of movements to the camera systems and treatment couch during the gantry rotation due to the considerable gantry mass of 670 tons, and since the SGRT camera systems are screwed directly to the gantry bearing. Additionally, the isocentric position deviation resulting from different couch angles was investigated. 3D laser tracker (FARO Technologies Inc, Lake Mary, FL) measurements were performed by positioning 3D markers on both camera systems and treatment couch, and tracking them (Figure 4). Translational and rotational position deviations of the markers related to the room isocentre point under gantry and couch angle 0° were determined three times. The results were compared to position deviations measured by AlignRT resulting from phantom movements using the static virtual human male CIRS pelvic phantom (CIRS, Norfolk, VA).

### 2.7 TPS‐based tool for SGRT

To avoid inefficiencies in the treatment planning, patient positioning and gated irradiation caused by various camera occlusions, for example, self‐occlusion by the patients, treatment head and setup devices, a RayStation TPS‐based tool was developed in our institution. Using this tool, obstruction of the camera systems can be detected early in the treatment planning process. Accordingly, suitable gantry and couch angles can be selected for treatment, allowing a sufficient area of the patient skin surface to be used for monitoring for gated irradiation during the RT course. This occlusion effect is reported in the literature and can be problematic for SGRT purposes.^40^, ^44^ The FOV of the tool was configured by means of the AlignRT calibration plate.^38^ The positions of the installed camera pods were measured relative to our room isocenter and provided to the tool. A S311 CPR Simon full body simulator (Gaumard Scientific, Miami, FL) was used to validate the reliability of the tool, and was positioned using a WingSTEP and knee cushion (Elekta, Stockholm, Sweden). A FOV comparison between the captures of all three SGRT systems at the gantry and the tool predictions in nine different planned isocenter points was performed.

### 2.8 Motion tracking accuracy of GateRT

To evaluate the motion tracking accuracy of GateRT, regular, and irregular respiratory patterns including different amplitudes and periods were applied using the CIRS phantom. The results were compared with the ground‐truth values (Table 1) to evaluate the temporal and spatial accuracy. As a benchmark for the comparisons of Cos^4^ breathing patterns, the fourth peak of the measured breathing curve (after a learning process in GateRT) was considered a stable match point to sync both the ground‐truth and measurement. For the regular and irregular breathing patterns, the peak of the maximum amplitude was used as a benchmark. The ground‐truth is the simulated breathing signal applied by the CIRS Phantom. For this purpose, a reference capture of the phantom including the sponge was performed, and a patch of 5 × 5 cm^2^ was created on this reference capture for recording the respiratory curve (Figure 1i). Measurements were performed under different gantry angles (between 270° and 90°) and with couch angle 0°. Different gantry and couch angles were used to show the effect of the gantry and couch angle dependency on measured breathing patterns. Various parameters of recorded breathing patterns were analysed: (i) Pearson correlation coefficient (PCC) to quantitatively evaluate the amplitude similarity between both breathing signals, ground‐truth and measurement, (ii) the mean absolute deviation (MAD) to quantitatively ascertain the discrepancy and reproducibility of paired observations (ground‐truth and measurement). In addition, the sampling rate of GateRT was also investigated.

Since the measurements were performed on different days, it was important to ensure that the phantom including the surrogate with the sponge measures the correct respiratory amplitude provided by the CIRS software. For all investigations, the same phantom setup, system settings of SimRT and GateRT were used including the same surrogate surface, ambient light and mid skin tone to have a consistent comparison under the same conditions (Figure 1).

### 2.9 Dosimetric verification

Irradiation plans including homogenous and heterogenous dose distributions with carbon ions were generated using the methods described in Section 2.4, and the clinical settings for patient plans in our institution. Heterogenous dose means that the target (chamber) moves within different iso‐dose regions with extremely steep dose gradients compared to the homogenous region.

Since the tumor target is moving, an ITV was generated from the CTVs contoured on the 3DCT datasets extracted from the 4DCT dataset. An ITV‐based median physical dose of 1 Gy was applied for both ion chamber and radiochromic film. For the treatment plans, the Raystation TPS, pencil‐beam dose engine, carbon ions, and dose grid settings of 1 mm were used. Two different gantry angles (30° and 90°), and only one couch angle 0° were used. The number of delivered energy layers varies between 9 layers for the film irradiation, 12 layers for the heterogenous plan, and 15 layers for the homogeneous plan. No repainting was performed, and a layer‐by‐layer raster‐scanning technique was used. Moreover, for each treatment plan, the dose was computed on each breathing phase in the determined gating window (Figure 3), phase‐weighted, and accumulated after applying a deformable image registration (DIR). In this context, dose accumulation involves merging individual doses derived from datasets that depict the anatomy at distinct respiratory phases, enhancing the precision of the intended dosage for the dynamically changing anatomy and resulting in a comprehensive cumulative dose.^52^ In simpler terms, dose accumulation introduces a temporal aspect to the traditional 3D dose representation.^52^ In this work, the biomechanically DIR of RayStation (Morfeus) was used.^54^, ^55^

In addressing the interplay effect mentioned in Section 1, commonly observed within the treated target and/or spared OARs due to the interplay between motion and scanning techniques, larger beam spots with a Full Width at Half Maximum (FWHM) of 10 mm were used in our treatment plans.

The following positioning workflow was applied to both experiments I.1 and I.2 (Figure 1e–i) prior to every measurement (ion chamber or film). First, the CIRS phantom was positioned on the 6 degrees of freedom robotic treatment couch^56^ by utilizing SGRT. Second, two planar kV images (Figure 2c–d) prior to the irradiation were acquired and matched to the digitally reconstructed radiographs which were generated from the projections of the planning CT series to a 2‐dimensional plane. After the kV matching using the immobile structures like bones (Figure 2c–d), rotational and translational correction vectors were calculated and applied to the couch to precisely position the phantom at the beam isocenter point(s). These applied correction vectors were compared with correction vectors in AlignRT.

In our work, we just investigated the phase‐based gating approach of GateRT. Whenever the position of the moving phantom is within the gating window, a beam‐on signal is sent automatically to our treatment machine for beam delivery. Otherwise, the beam is held (Figure 3).

#### 2.9.1 Absolute dosimetry

The PinPoint chamber 0.03 cm^3^ (Type 31015)^57^ (PTW, Freiburg, Germany) was used to perform quantitative point dose measurements. For this aim, a lung‐tissue equivalent rod including an insert for the PinPoint chamber (Figure 1e–i) was used which includes a spherical target with a diameter of 3 cm in which a PinPoint chamber was placed at the tumor center (Figure 2a,c–b). A two‐beams plan with gantry angles 30° and 90° was applied. Further, the ionization chamber was pre‐irradiated with 2 Gy. The measured dose was calculated and corrected according to the following formula^15^, ^58^:

(2) $$D_{w}\left(P_{eff}\right)=M_{Corr}\cdot N_{D,\;w,\;Co-60}\cdot K_{Q}$$

where M_Corr_ is the dosimeter reading M, corrected for changes in air density, incomplete saturation, and polarity effects of the chamber. The calibration factor, N_D, w, Co‐60_, is given by PTW and k_Q_ is a chamber‐specific factor that corrects for the different beam quality of carbon ions with respect to the calibration beam quality (^60^Co). The irradiation of homogenous and heterogenous plans was performed three times for each following situation: (i) static at 0% inhale phase (without tumor motion), (ii) non‐gated with motion, and (iii) gated with motion in the same manner. The mean and standard deviation (SD) of the three irradiations were calculated and compared with the TPS reference dose in the homogeneous dose region of each plan.

#### 2.9.2 Relative film dosimetry

For symmetry and uniformity tests, radiochromic films Gafchromic (EBT3)^59^, ^60^ (International Specialty Products, Wayne, NJ) were irradiated using a one‐beam plan with a gantry angle of 90° (Figure 1e). Three measurements were performed: (i) static, (ii) non‐gated, and (iii) gated. The radiochromic film insert of CIRS is designed to hold the film at the sagittal cross‐section along the long axis (Figure 2b,e). The beam scan direction during the irradiation was IS. After the film irradiation, the film blackening along the motion axis (IS) was evaluated using Verisoft (PTW, Freiburg, Germany). The flat‐bed Epson Perfection V750 Pro scanner (EPSON, Suwa, Japan) in the transmission mode using a spatial resolution of 150 dpi was used, and all films were analyzed in the red channel of 48‐bit RGB.^60^, ^61^, ^62^

The following geometric parameters were evaluated: (i) FWHM, (ii) the width of the lateral fall‐off from 80% to 20%, (ii) the width of the radiation field of 90%, and (iv) the shift of dose delivery caused by gating irradiation. The latter was determined by calculating the difference in the blackened segments on the films at FWHM, and compared to the shift calculated in TPS. Both the static and gated measurements were aligned by using beam spots which were irradiated under static conditions. Shifts of dose distributions obtained using gating were reported in previous studies and guidelines for both photon and particle therapies,^40^, ^44^, ^45^, ^63^, ^64^, ^65^, ^66^, ^67^ and identified to be caused by delay times. Such a delay time in beam on/off can result in an under‐ and overdosage of OAR.^66^ The shift (∆L) can be converted to a time delay (T_d_), using the known velocity of the moving radiochromic film rod inside the lung by $T_{d}=\frac{\Delta L}{V}$ (Figure 3).

## 3 RESULTS

### 3.1 Gantry and couch angle dependency

**FIGURE 5:**
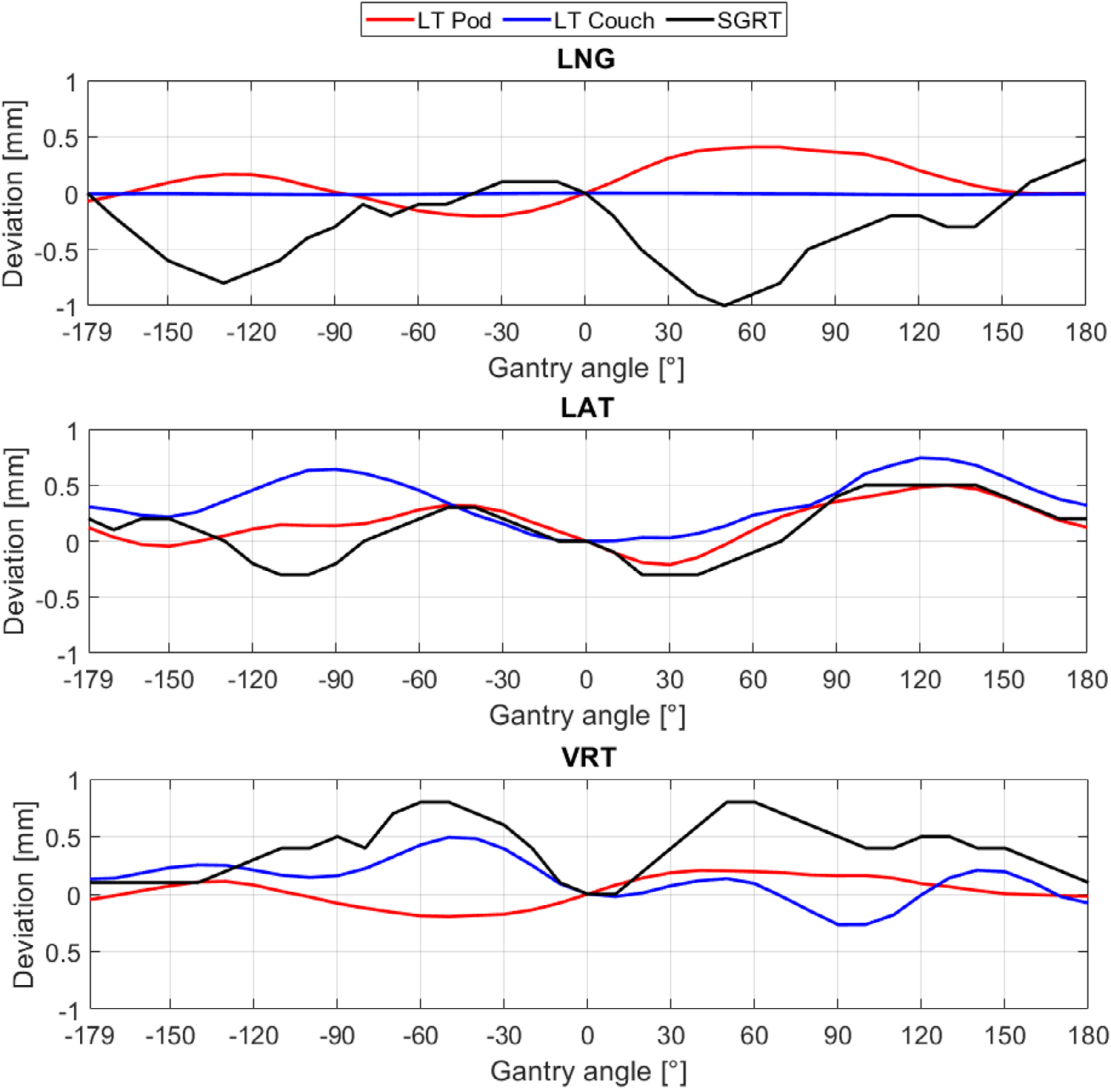
Translational deviations caused by the ion beam gantry angle dependency for treatment couch, camera pods measured by laser tracker, and for SGRT measured by a pelvis phantom. Gantry was moving counterclockwise. LAT, lateral; LNG, longitudinal; LT, laser tracker; SGRT, surface‐guided RT; VRT, vertical.

Figure 5 shows the gantry angle dependency as well as its influence on the treatment couch and camera pods while rotating the gantry using the laser tracker. These results are compared with phantom measurements performed by AlignRT.

The translational deviations (mean ± SD) are (i) 0.10 ± 0.19 mm, 0.16 ± 0.19 mm, 0.03 ± 0.12 mm for the camera Pods, (ii) −0.01 ± 0.00 mm, 0.35 ± 0.23 mm, 0.12 ± 0.18 mm for the couch, and (iii) −0.31 ± 0.34 mm, 0.11 ± 0.26 mm, 0.39 ± 0.24 mm for the SGRT in longitudinal (LNG), lateral (LAT), and vertical (VRT) directions, respectively. The rotational deviations for both laser tracker and SGRT are negligible with a maximum deviation of −0.01 ± 0.01° (Figure S2).

**FIGURE 6:**
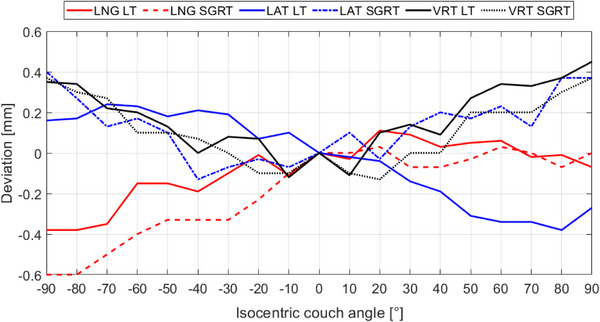
Translational deviations caused by the couch angle dependency measured by laser tracker, and compared with SGRT using a pelvis phantom. LAT, lateral; LNG, longitudinal; LT, laser tracker; SGRT, surface‐guided RT; VRT, vertical.

Figure 6 and Figure S3 depict the couch angle dependency using the laser tracker and AlignRT. The translational deviations (mean ± SD) are (i) −0.08 ± 0.15 mm, −0.03 ± 0.22 mm, 0.17 ± 0.17 mm for the laser tracker, and (ii) −0.19 ± 0.22 mm, 0.13 ± 0.16 mm, 0.11 ± 0.17 mm for AlingRT in LNG, LAT, and VRT, respectively. The rotational deviations for both laser tracker and SGRT are also negligible with a maximum deviation of 0.04 ± 0.1°.

### 3.2 TPS‐based tool for SGRT

**FIGURE 7:**
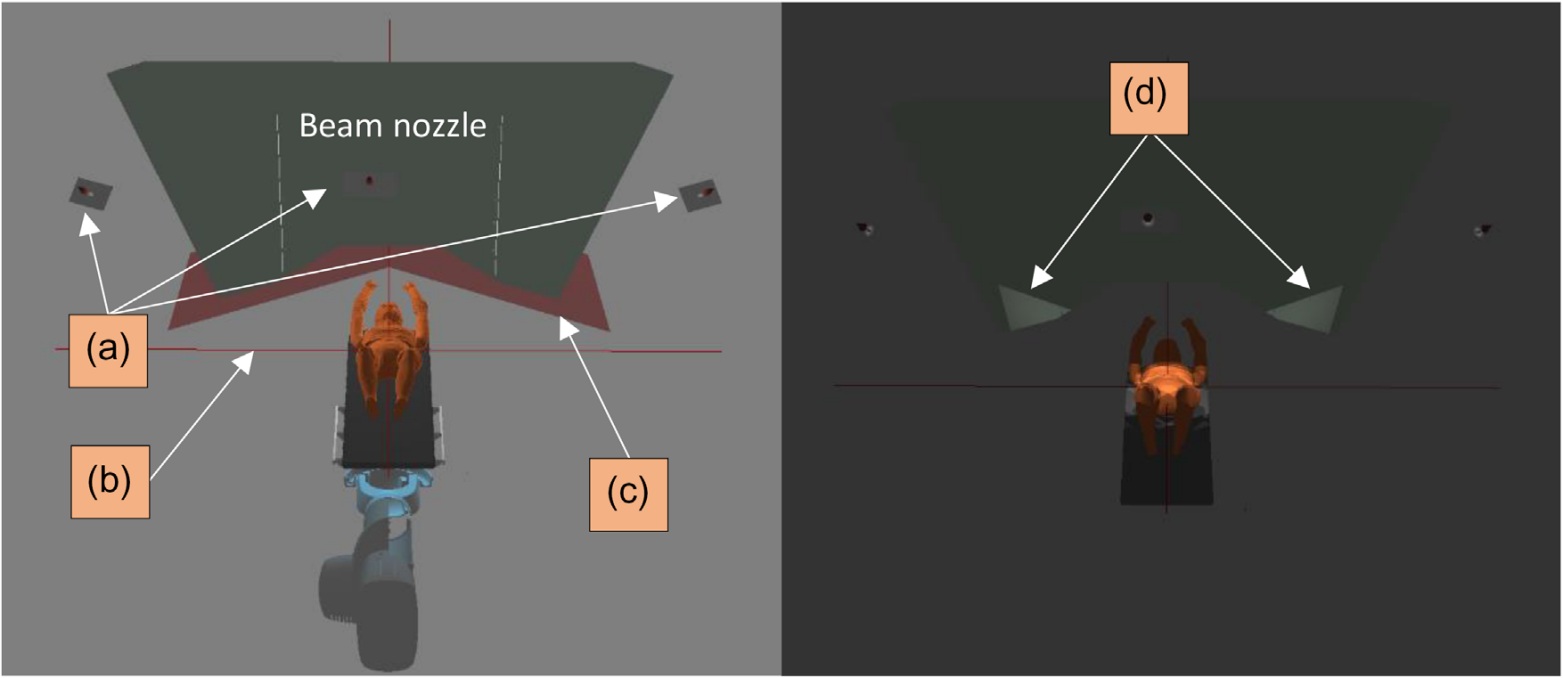
Left image illustrates the components configured in the tool: beam nozzle, reference skin surface of the phantom (in orange), camera pods (a), room isocentre (b), and the region of laser scanner which are used to detect collisions between the beam nozzle, treatment couch and any other setup devices (c). Right image illustrates the camera occlusion caused by the beam nozzle (d). Gantry is under 0° in this image.

**FIGURE 8:**
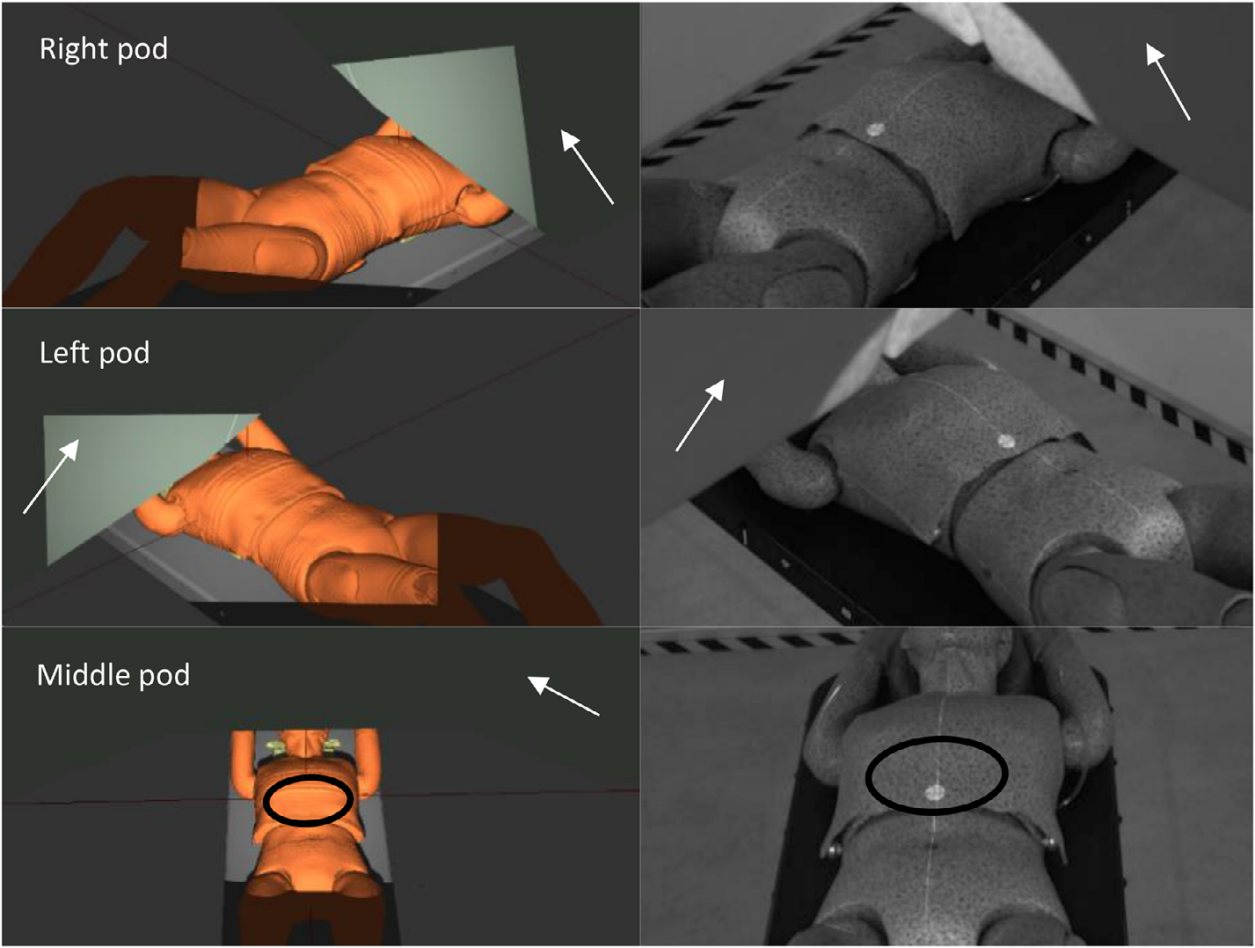
Left images show the different FOVs of the configured camera pods in the TPS (right, left, and middle pod) displaying the body region (black circle on the abdomen) needed for gating. Right images illustrate the real FOVs captured by AlignRT. Note that the tool is able to show the shadow caused by the patient, setup devices, and beam nozzle (white arrows). FOV, field of view; TPS, treatment planning system.

Figure 7 displays the TPS‐based tool including the room isocenter, beam nozzle, treatment couch, and camera pods. The tool verification using a full body simulator was performed using a treatment plan with different gantry, couch angles, and in different body regions. Figure 8 shows three comparisons between the FOV provided by the TPS‐based tool on the left side, and the real captures provided by AlignRT on the right side.

### 3.3 Motion tracking accuracy of GateRT

Table 3 presents the different results of all measured respiratory patterns. By comparison with the ground‐truth under gantry angle 0°, GateRT shows peak‐to‐peak amplitude differences between 0.03 ± 0.02 mm and 0.26 ± 0.03 mm for all investigated respiratory patterns, whilst the periods ranged with a SD between 10 and 190 ms. Furthermore, all respiratory patterns of the volunteer and cos^4^ were well reproduced with a maximal MAD of 0.18 ± 0.15 mm, 0.23 ± 0.19 mm, and 0.30 ± 0.23 mm for C, RV and IRV patterns, respectively. Table 3 also depicts the relationships between the ground‐truth and measurements by GateRT using PCC assessments. The results obtained with GateRT show a high PCC of 0.998−1.000 for all respiratory patterns.

**TABLE 3:** Summary of peak‐to‐peak amplitudes and MADs between the ground‐truth and measurements recorded by GateRT for all regular and irregular respiratory patterns.

Gantry 0°				
Respiratory pattern	A ± SD [mm]	T ± SD [s]	MAD ± SD [mm]	PCC
C1	1.97 ± 0.02	2.00 ± 0.02	0.05 ± 0.03	0.998
C2	1.97 ± 0.02	4.00 ± 0.07	0.04 ± 0.03	0.999
C3	7.74 ± 0.03	2.00 ± 0.03	0.18 ± 0.15	0.998
C4	7.86 ± 0.04	4.00 ± 0.05	0.08 ± 0.08	1.000
C5	15.77 ± 0.07	2.00 ± 0.03	0.15 ± 0.13	1.000
C6	15.80 ± 0.05	3.99 ± 0.06	0.15 ± 0.14	1.000
RV1	10.23 ± 1.13	7.43 ± 0.54	0.18 ± 0.18	1.000
RV2	16.21 ± 0.90	6.79 ± 0.21	0.23 ± 0.19	1.000
RV3	17.95 ± 2.10	7.71 ± 0.54	0.14 ± 0.13	1.000
RV4	20.63 ± 1.59	6.30 ± 0.24	0.17 ± 0.17	1.000
IRV1	15.81 ± 2.71	9.39 ± 3.49	0.21 ± 0.19	0.999
IRV2	14.07 ± 3.77	5.90 ± 1.69	0.28 ± 0.21	0.999
IRV3	14.36 ± 3.35	5.41 ± 1.08	0.17 ± 0.11	1.000
IRV4	14.22 ± 6.69	5.10 ± 2.35	0.30 ± 0.23	0.999

*Note*: The Pearson correlation coefficients between the respiratory patterns are also presented. Mean and SD of the entire breathing signal are presented.

Abbreviations: A, amplitude; C, cos^4^; IRV, volunteer with irregular breathing; MAD, mean absolute deviation; PCC, Pearson correlation coefficient; RV, volunteer with regular breathing; SD, standard deviation; T, period.

Table 4 presents the influence of gantry angle dependency on the recorded Cos^4^ respiratory patterns under different gantry angles. The maximal amplitude difference between the measurements and the ground‐truth is slightly bigger than in Table 3. However, the PCC and T values are in the same range.

**TABLE 4:** Summary of peak‐to‐peak amplitudes and MADs between the ground‐truth and measurements recorded by GateRT for Cos^4^ respiratory patterns.

Respiratory pattern C6				
Gantry angle [°]	A ± SD [mm]	T ± SD [s]	MAD ± SD [mm]	PCC
270	15.76 ± 0.07	4.00 ± 0.05	0.19 ± 0.19	0.999
300	15.68 ± 0.06	3.99 ± 0.06	0.33 ± 0.28	0.998
330	15.69 ± 0.06	4.01 ± 0.04	0.15 ± 0.13	1.000
30	15.67 ± 0.09	4.00 ± 0.06	0.16 ± 0.13	1.000
60	15.68 ± 0.05	4.00 ± 0.03	0.16 ± 0.15	1.000
90	15.74 ± 0.09	4.00 ± 0.07	0.12 ± 0.12	1.000

*Note*: The Pearson correlation coefficients between the respiratory patterns are also presented. Note that the reference capture was taken under gantry angle 0°. Mean and SD of the entire breathing signal are presented.

Abbreviations: A, amplitude; C6, cos^4^ (A = 16 mm, T = 4 s); MAD, mean absolute deviation; PCC, Pearson correlation coefficient; SD, standard deviation; T, period.

**FIGURE 9:**
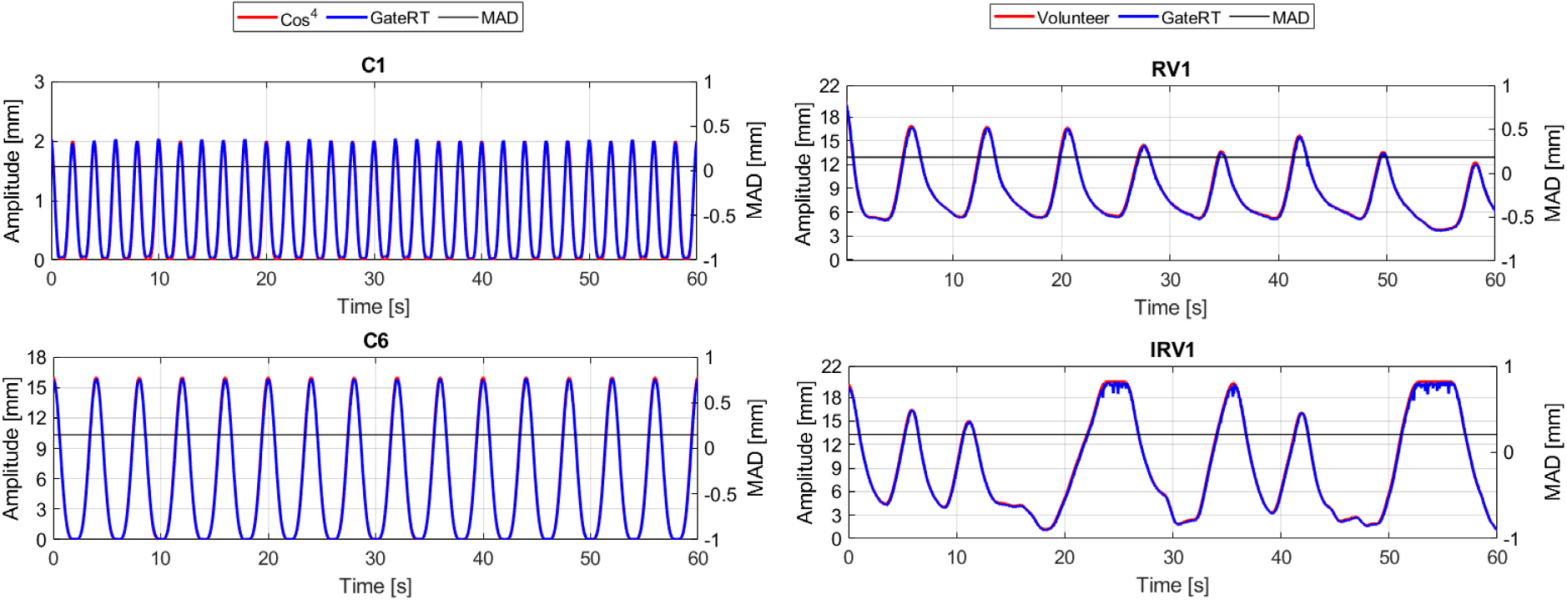
Four respiratory patterns measured by GateRT under gantry angle 0° using the CIRS phantom compared with the ground‐truth. A, amplitude; C1, cos^4^ (A = 2 mm, T = 2 s); C6 = cos^4^ (A = 16 mm, T = 4 s); IRV, volunteer with irregular breathing; MAD, mean absolute deviation; RV, volunteer with regular breathing; T, period.

Figure 9, Figure S4–S6 display all investigated respiratory patterns under different gantry angles presented in the time domain. The results of the measured respiratory patterns confirm the strong correlation between the GateRT and ground‐truth. Moreover, the sampling rate of GateRT varies during one measurement. Figure S7 illustrates a histogram of all sampling rates recorded during one measurement with a mean value of 22.7 ± 0.5 Hz.

### 3.4 Absolute dosimetry

Table 5 compares the absolute dose deviations between measurements (static, non‐gated, gated) and calculations (TPS). On the one hand, the respiratory‐gated irradiation presents a dose deviation of 3.50 ± 0.70% for the homogenous plan versus TPS, while 0.10 ± 4.01% were resulted for the heterogenous plan. On the other hand, the non‐gated measurements were the worst with 11.77 ± 7.24% and 27.55 ± 13.90% for homogenous and heterogenous plans, respectively.

**TABLE 5:** Results of absolute dose deviations between TPS calculations and measurements.

	Absolute dose deviation Mean ± SD [%]		
Dose distribution	TPS_Stat_—Measurement_Stat_	TPS_Gated_—Measurement_Gated_	TPS_Stat_—Measurement_non‐gated_
Homogenous dose	3.15 ± 0.23	3.50 ± 0.70	11.77 ± 7.24
Heterogenous dose	5.00 ± 0.13	0.10 ± 4.01	27.55 ± 13.90

*Note*: Positive numbers mean that the measurement was smaller than the TPS calculation. Mean and SD of three measurements are presented. Non‐gated means tumor motion without gating, and static means no motion.

Abbreviations: SD, standard deviation; Stat, static; TPS, treatment planning system.

To show the effect of respiratory irregularities during the phase‐based gating irradiation, the respiratory peak‐to‐peak amplitude of the surrogate was increased from 16 to 25 mm. Accordingly, a dose deviation of −47 ± 1.3% was achieved.

Additionally, the discrepancy between AlignRT and plan kV imaging was calculated to assess the positioning accuracy of AlignRT. So, the deviations (mean ± SD) of LAT, LNG, VRT, Yaw, Roll, and Pitch for all dosimetric measurements were 0.27 ± 0.30 mm, 0.63 ± 0.31 mm, −0.57 ± 0.16 mm, 0.13 ± 00.00°, 0.03 ± 0.13°, −0.17 ± 0.04°, respectively. It should be considered that the final phantom positioning was performed by the planar kV imaging.

### 3.5 Relative film dosimetry

**FIGURE 10:**
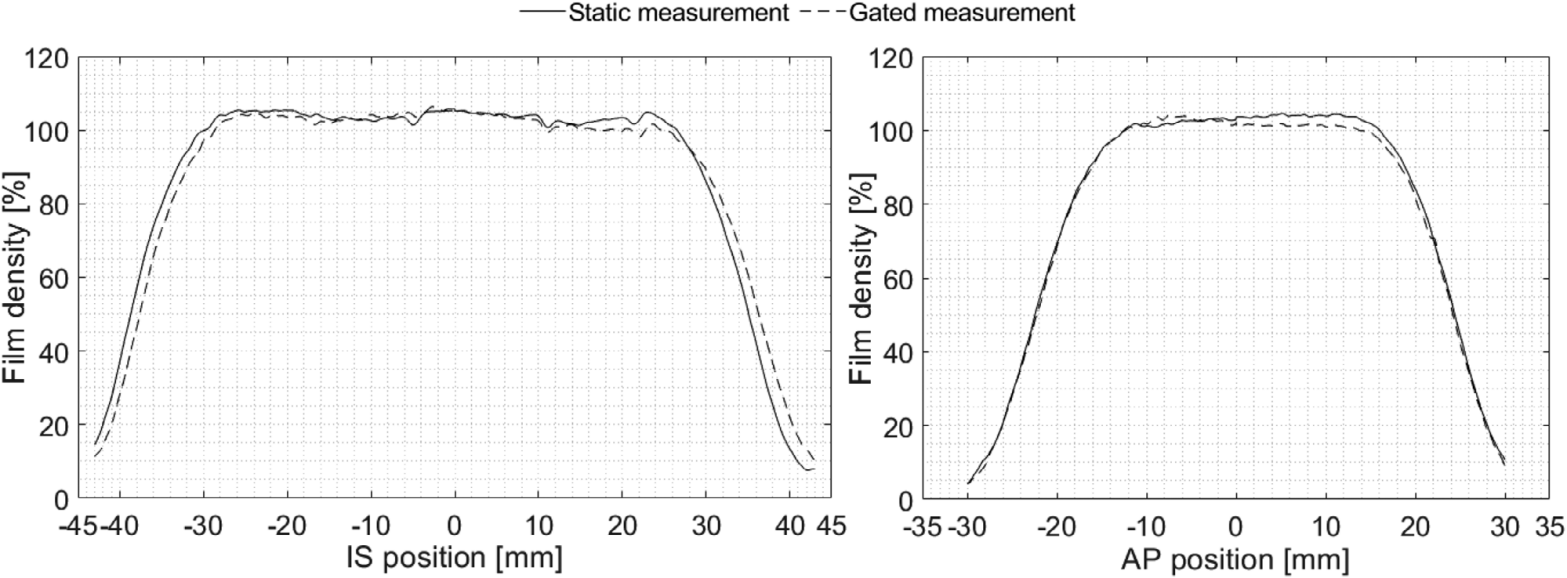
Relative dose profiles measured on the EBT3 films at both planes IS and AP with Cos^4^ motion (peak‐to‐peak amplitude = 20 mm, period = 4 s). IS profiles show the direction of the moving films (scan direction). Mean and SD of three measurements are presented. AP, anteroposterior; IS, inferior‐superior; SD, standard deviation.

The motion during the gate‐on period (2.12 s, 53% of one period) was 6 mm (30% of peak‐to‐peak amplitude). Table 6 shows the geometrical evaluation of the relative dose profiles presented in Figure 10. The FWHM, field size, and penumbra values of the gated measurements agree within 1 mm with the static measurements for both IS and AP profiles. The beam on/off shifts presented in Table 6 were only found in the IS scan direction, and comparable with shifts calculated in the TPS within 0.13 mm. The beam on/off delay times were approximately 50.98 ± 9.85 ms and 54.91 ± 7.82 ms, respectively.

**TABLE 6:** Geometrical evaluation of relative dose profiles measured on EBT3 films using static and gated measurements.

	Static measurement—gated measurement	
	IS Mean ± SD [mm]	AP Mean ± SD [mm]
FWHM	−0.41 ± 0.37	0.18 ± 0.27
Field size at 90%	0.73 ± 0.87	0.48 ± 0.25
Left lateral fall‐off 80% to 20%	0.59 ± 0.38	−0.01 ± 0.28
Right lateral fall‐off 80% to 20%	−0.05 ± 0.19	−0.19 ± 0.18
Beam‐On shift at 50%	−1.04 ± 0.20	−0.20 ± 0.18
Beam‐Off shift at 50%	−1.12 ± 0.16	−0.13 ± 0.04

Abbreviations: AP, anteroposterior; FWHM, full width at half maximum; IS, inferior‐superior; SD, standard deviation.

## 4 DISCUSSION

In this work, a surface‐guided E2E test of phase‐based gating using the GateRT system was presented for ion beam RT in dynamic phantoms. To achieve precise gated irradiation, the entire clinical workflow under clinical conditions was investigated, including planning CT simulation, treatment planning, positioning accuracy, gated irradiation, and dosimetric assessment. In the literature, several external RMSs for gated irradiations were investigated such as Varian Position Management system (RPM, Varian, CA), Anzai belt (Anzai Medical Co., Ltd., Shinagawa, Tokyo), SDX spirometer (Muret, france), CPX spirometer (Medgraphics, St. Paul), Abches (Yamanashi, Japan), and Catalyst (C‐Rad, Upsalla, Sweden). However, there are no studies investigating the GateRT which has recently been updated to its new version “Respiratory Module”. Thus, the methods presented in this work could be re‐used for evaluating the announced free‐breathing gating feature in AlignRT “Respiratory Module”, which will replace GateRT in the near future.

### 4.1 Gantry and couch angle dependency

First tests investigating the gantry and couch angle dependency show that the positioning and tracking accuracy of the camera pods are affected by the applied gantry and couch rotation. These systematic sub‐millimeter deviations are within tolerances provided by AAPM task group^40^ and ESTRO‐ACROP.^44^

For our respiratory‐induced gating purposes such small deviations can be neglected as a new reference capture at the desired gantry and/or couch angle will be captured by GateRT, thus accounting for this systematic offset. For positioning purposes where the reference surface from the planning CT is used, such a small deviation of <1 mm is still acceptable.

### 4.2 Motion tracking accuracy of GateRT

Further experiments assessing the motion tracking accuracy and reproducibility of regular and irregular breathing patterns indicate a stable and high real‐time breathing detection of amplitude and period with a maximum SD of 0.15% and 4.75%, respectively, compared to ground‐truth. The amplitude deviations are within the tolerance of 1 mm provided by ESTRO‐ACROP.^44^ Moreover, a strong correlation of the investigated breathing patterns of approximately 1 compared to the ground‐truth in all measurements was observed, thus resulting in an accurate spatial and temporal motion reconstruction for gating purposes.

Our results also indicate that the sampling rate of GateRT position detection varies within a single measurement, potentially resulting in incorrect peak and valley detection, especially when smaller periods are applied. Smaller periods are applied if the patients breathe faster. Such variations may lead to inaccuracies in dose delivery which is based on the detection accuracy of the peak and valley values. Consequently, the treatment quality can be compromised. Despite the observed variations in our work, GateRT precisely reproduced the given breathing patterns.

In order to assess the absolute static positioning accuracy of AlignRT, an optical‐radiographic comparison was conducted, AlignRT against planar kV imaging. All observed translational and rotational discrepancies were found to fall within sub‐millimeter and degrees ranges, thereby meeting the tolerances stipulated by AAPM Task Group^40^ and ESTRO‐ACROP.^44^

### 4.3 Absolute dosimetry

Commissioning of respiratory‐induced gating systems should cover an assessment of both radiation dosimetry accuracy (including geometrical analysis of irradiated beams), and beam on/off performance (latency time) in comparison to static irradiations.

Our investigations show absolute dose deviations within our institutional tolerances of ±5% mean value and ±7% minimum and maximum values. As compared to static irradiation, the absolute dose SD of the heterogenous gated irradiation depicts a high value of ±4% which were less pronounced for the homogenous plan, since the heterogenous plan was designed with steeper dose gradients closer to the chamber (measurement position). Hence, the plan was more sensitive regarding such steeper dose gradients and position uncertainty. However, both irradiation plans were clinically acceptable.

### 4.4 Relative film dosimetry

The reliability of GateRT was also assessed by the irradiated EBT3 films, and showed a relatively precise dose distribution, reflecting an acceptable geometry on a moving phantom in both planes AP and IS (scan direction). Geometrical parameters of AP profiles were within ± 0.5 mm, whilst the IS profiles provide slightly greater mean deviations but still less than 1 mm. All geometrical deviations are within our institutional tolerances of 1.5 mm.

Figure S8 illustrates the blackening on the films (static, non‐gated, and gated), and the resulted dose conformity can be also seen. Moreover, no interplay effects were seen in form of over‐ or underdosage by the gated irradiation, compared to the non‐gated irradiation where the effect is clearly depicted.

**FIGURE 11:**
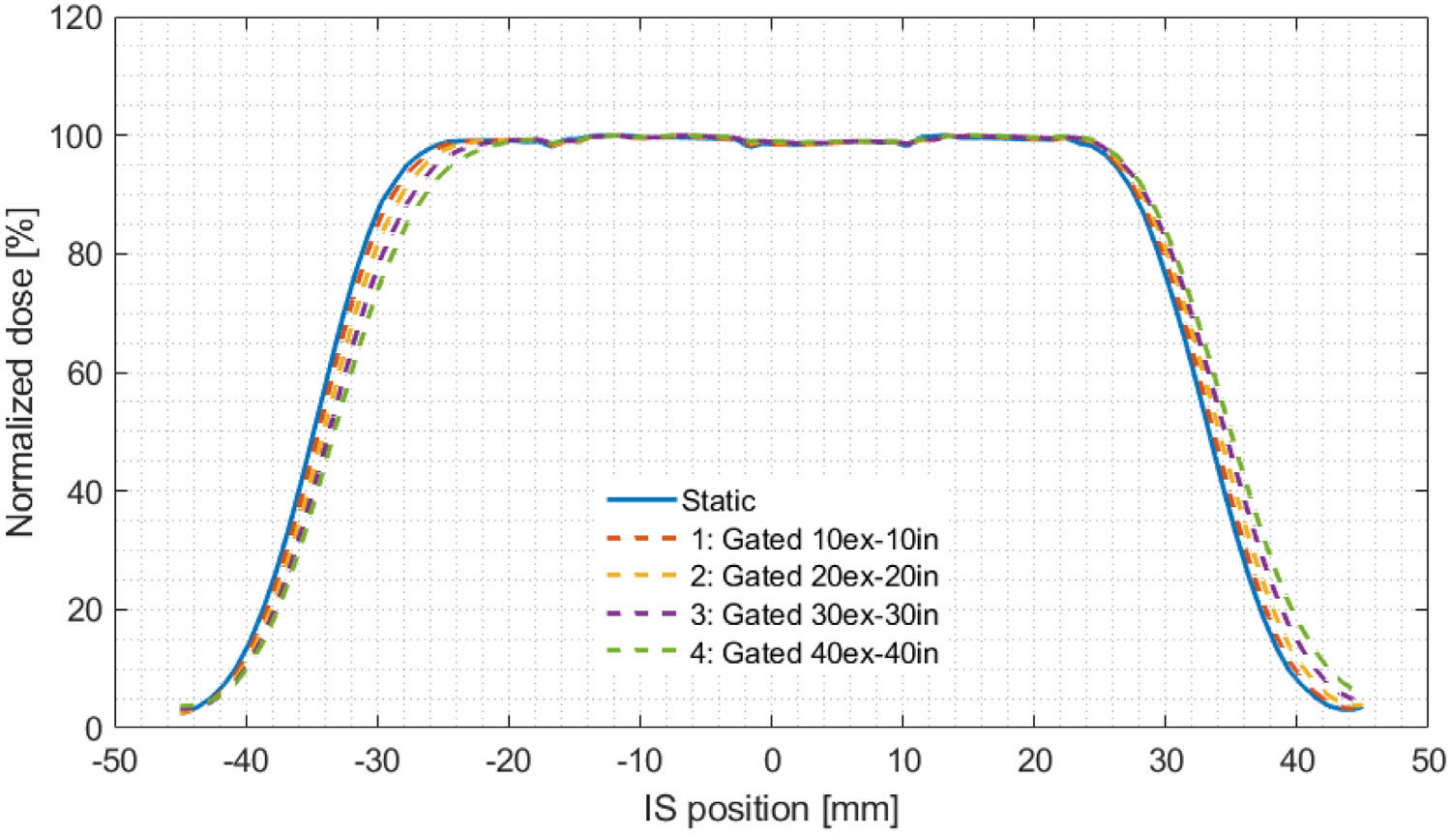
Extracted profiles from TPS showing the dependency between the gating window and the dose distribution shift in the direction of moving films. Resulted beam‐on shifts are −0.35, −0.76, −1.17, −1.56 mm for gating windows 1, 2, 3, and 4, respectively, and for beam‐off −0.34, −0.78, −1.23, −1.67 mm for gating windows 1, 2, 3, and 4, respectively. ex, exhale; in, inhale; IS, inferior‐superior; TPS, treatment planning system.

The last crucial parameter investigated in this study was the time delay of beam on/off which usually is expected when using gated irradiations. For the regular breathing pattern Cos^4^ with amplitude of 20 mm and period of 4 s, the overall time delays were less than 60 ms and thus within both recommended tolerances from ESTRO^44^ and AAPM^40^, ^45^ of 200 and 100 ms, respectively. A variety of factors may contribute to the delay time such as data acquisition, processing, and communication between the gating interface and treatment machine.^67^ The beam shift caused by this delay time agreed with the TPS prediction presented in Figure 11 (Gated 30ex‐30in) which also presents other beam shifts for different gating windows.

Furthermore, we found a relationship between the gating window and the resulted beam shift (Figure 11) which depends on the velocity of the tumor motion. That being said, flatter breathing curves provide smaller beam shifts. In our case, the gating window was placed around the end‐expiration phase, and reducing the gating window on both sides equally (i.e. including more breathing phases with less motion velocity) minimizes the remaining beam shift. Consequently, more normal tissue may be spared.

However, the time of gated treatments should be considered because selecting smaller gating windows prolongs the treatment time. Vedam et al.^68^ reported that the latter advantage of sparing OARs may be eliminated by inter‐fractional errors. Besides, prolonged treatments may be not comfortable for the patients, resulting in breathing irregularities.^66^ Nevertheless, all investigated gating windows in this work resulted in delay times less than 100 ms, which is clinically acceptable. That being said, the beam shift can vary based on the shape of the breathing pattern, breathing period, and location of the gating window. Consequently, it should be considered in ITV margin calculations. Additionally, variations in period or/and amplitude (i.e. breathing irregularities) should also be considered during the treatment irradiation, for example, by providing the patient with a visual coaching that they can reproduce their breathing signal regularly.

### 4.5 TPS‐based tool for SGRT

Using the TPS‐based tool, the most suitable planning scenarios can be predicted, thus making sure sufficiently large portions of the skin surface can be used for patient positioning and gating. Additionally, less occlusion of the camera systems by the treatment machine can be achieved, including range shifter, gantry angle, couch position and patient setup. Such simulations can improve the planning and workflow efficiency, hence saving time and improving the treatment quality, especially in such a more complicated treatment room construction than conventional linacs.

## 5 CONCLUSIONS

In conclusion, a comprehensive E2E test including the performance, reliability, and quality of the gated irradiation at an ion beam gantry was performed before implementing it into the clinical workflow. Three commercial SGRT modules (SimRT, AlignRT, and GateRT) were involved, starting with 4DCT image acquisition by SimRT, followed with phantom positioning by AlignRT, and gating by GateRT.

Based on phantom measurements, our Results show a clinically‐appropriate spatial and temporal accuracy of the three investigated systems in clinical applications, when comparing with international and institutional guidelines and tolerances, and without placing any physical devices on the patient skin which can affect the water equivalent thickness of ions.^69^ Further, the dosimetric accuracy and beam shape characteristics were determined to be clinically appropriate and within our institutional tolerances. It is also advisable to gate at phases that are more stable and reproducible.

Finally, it is imperative to account for the limitations of SGRT as outlined in international guidelines as thermal drift, skin tone, and FOV occlusion.^40^, ^44^ A quality assurance program should be established based on recommendations from AAPM and ESTRO, adapted to motion management in particle therapy, and our institutional needs before integrating the system for clinical uses.

## AUTHOR CONTRIBUTIONS

Abdallah Qubala designed the research study, analyzed the data, and wrote the manuscript. Jehad Shafee conducted experiments, and analyzed the data. Thomas Tessonnier provided technical expertise and guidance throughout the study, and reviewed the manuscript. Julian Horn provided technical expertise throughout the study, and reviewed the manuscript. Marcus Winter provided technical expertise and guidance throughout the study, and approved the final version for publication. Jakob Naumann assisted with data analysis, and reviewed the final version of the manuscript. Oliver Jäkel provided critical feedback on the manuscript, and approved the final version for publication.

## CONFLICT OF INTEREST STATEMENT

The authors declare no conflicts of interest.

## Supporting information

Supplementry information

## Data Availability

Research data are stored in an institutional repository and will be shared upon request to the corresponding author.
