# Supplementary material for: Characteristics of breathing‐adapted gating using surface guidance for use in particle therapy: A phantom‐based end‐to‐end test from CT simulation to dose delivery
Source: J Appl Clin Med Phys. 2023 Dec 21;25(1):e14249. doi: 10.1002/acm2.14249 (PMC10795430; doi:10.1002/acm2.14249)
Supplement: Supplementary file 1 — Supplementry information [file ACM2-25-e14249-s001.docx]

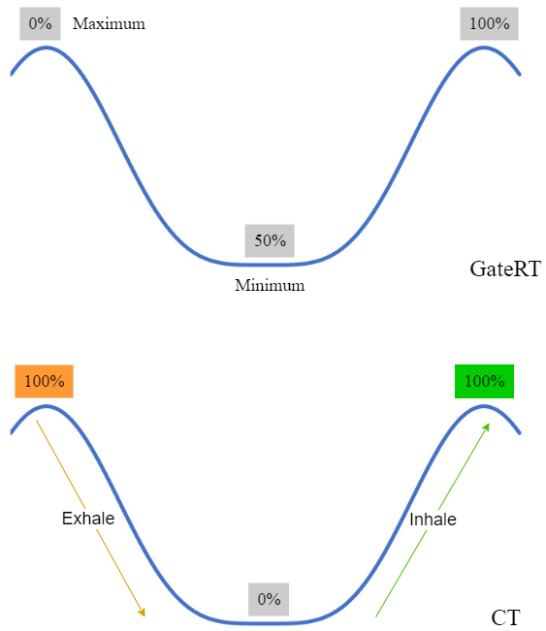


Figure S1: The difference of percentage phase fragmentation by the CT scanner and GateRT of a respiratory cycle. 20% inhale or exhale in the CT curve corresponds to 10% inhale or exhale in the GateRT curve.


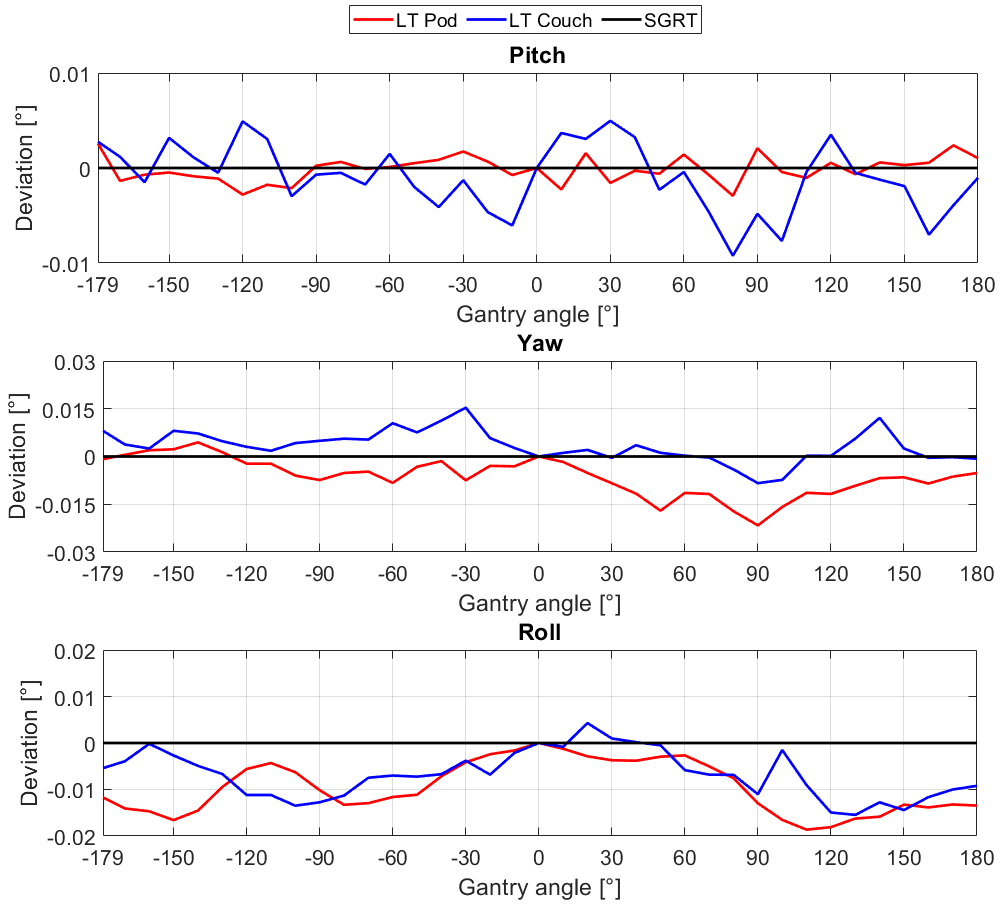


Figure S2. Rotational deviations caused by the ion beam gantry angle dependency for treatment couch, camera pods measured by laser tracker, and for SGRT measured by a pelvis phantom. Gantry was moving counterclockwise. Abbreviations: LT = laser tracker.


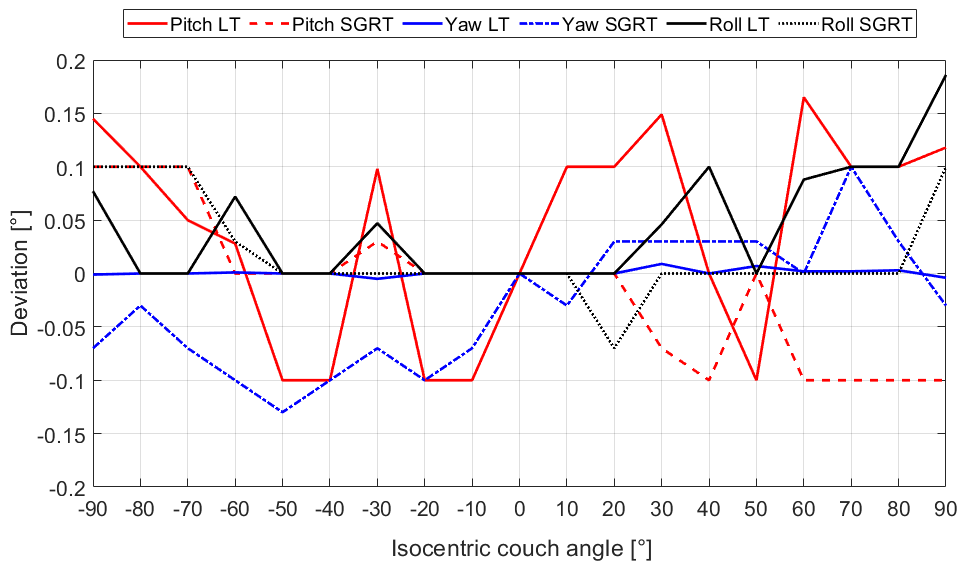


Figure S3. Rotational deviations caused by the couch angle dependency measured by laser tracker, and compared with SGRT using a pelvis phantom. Abbreviations: LT = laser tracker; LAT = lateral; LNG = longitudinal; VRT = vertical.


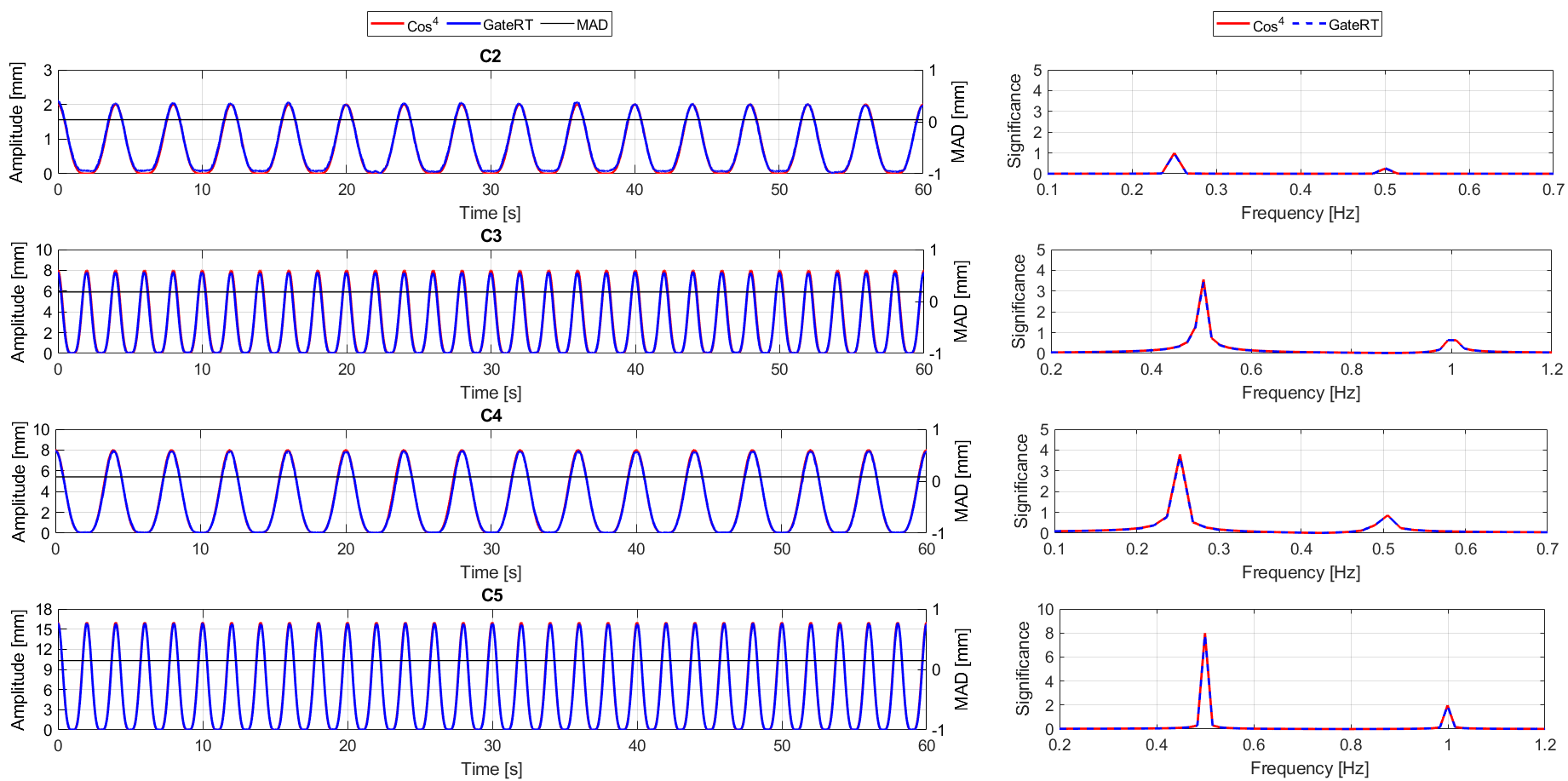


Figure S4. Respiratory patterns measured by GateRT using the CIRS phantom under gantry angle 0° compared with the ground-truth. Abbreviations: A = amplitude; C2 = cos^4^ (A = 4 mm, T = 2 s); C3 = cos^4^ (A = 4 mm, T = 4 s); C4 = cos^4^ (A = 8 mm, T = 2 s); C5 = cos^4^ (A = 8 mm, T = 4 s); MAD = mean absolute deviation; T = period.


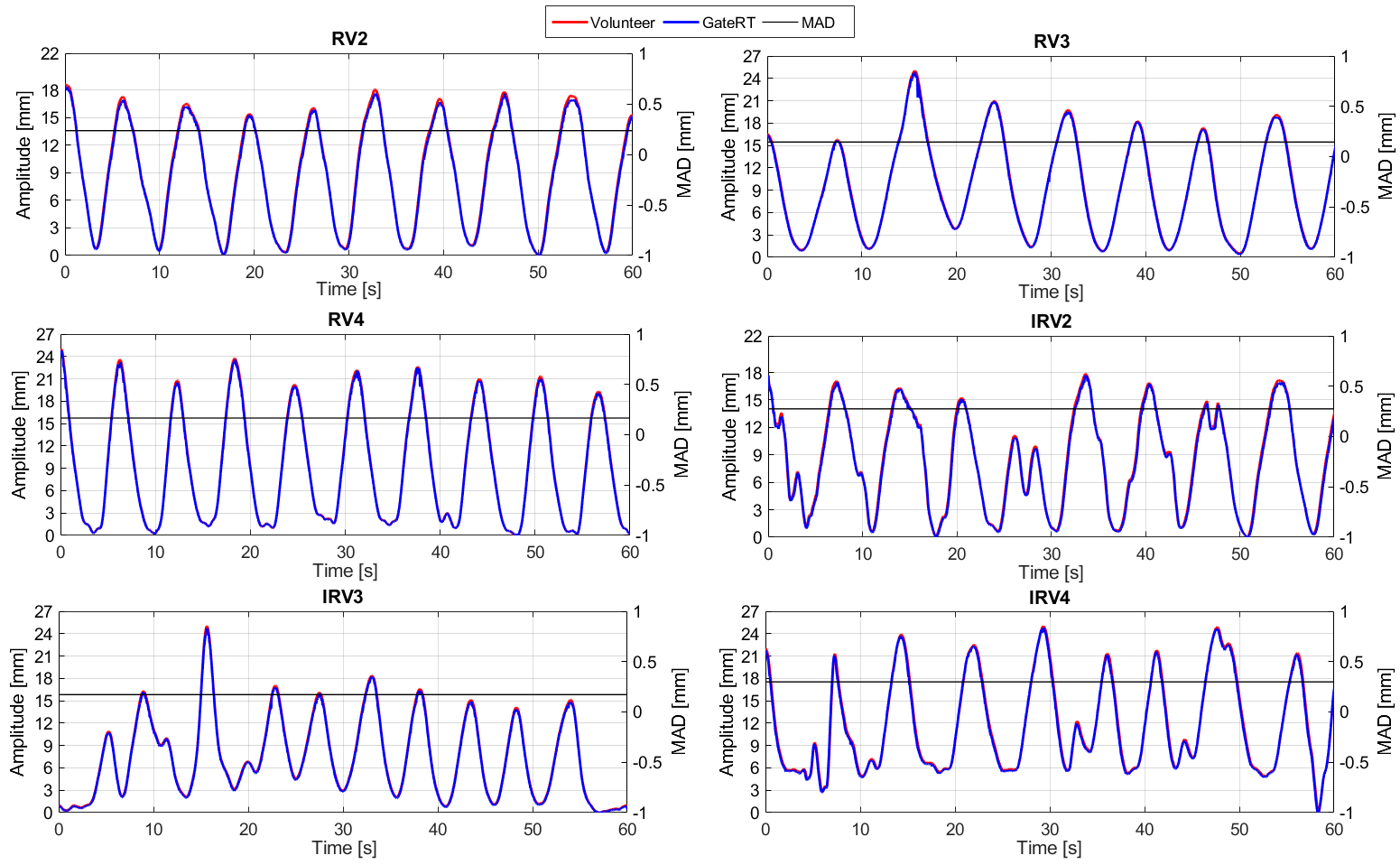


Figure S5. Respiratory patterns measured by GateRT using the CIRS phantom under gantry angle 0° compared with the ground-truth. Abbreviations: IRV = volunteer with irregular breathing; MAD = mean absolute deviation; RV = volunteer with regular breathing.


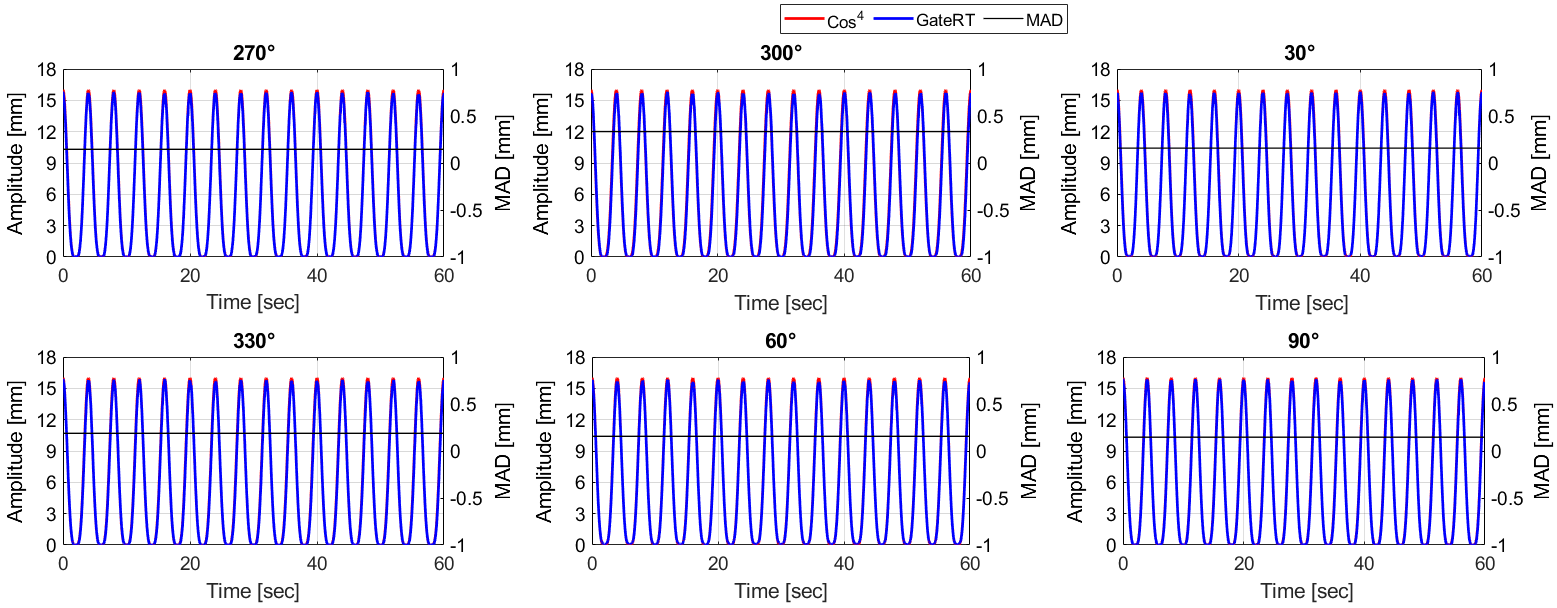


Figure S6. Cos^4^ respiratory patterns measured by GateRT using the CIRS phantom under different gantry angles compared with the ground-truth. Note that the reference capture was taken under gantry angle 0°. Abbreviations: MAD = mean absolute deviation.


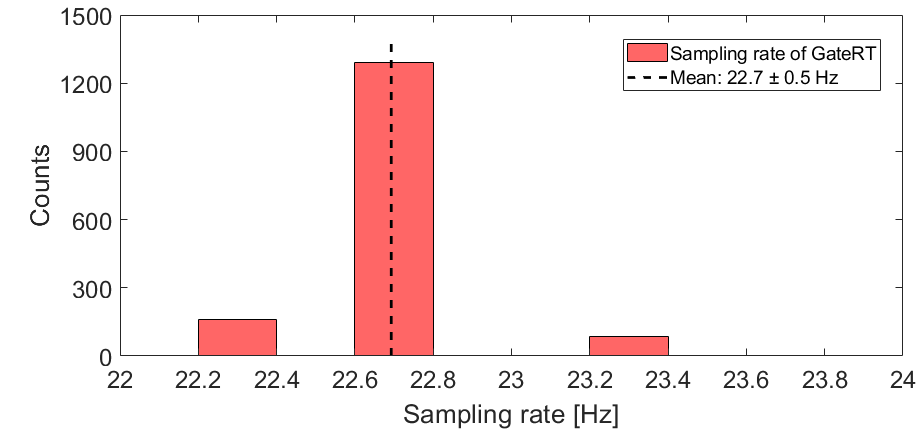


Figure S7. Distribution of the GateRT sampling rate for one measurement. The mean sampling rate is 22.7±0.50 Hz.


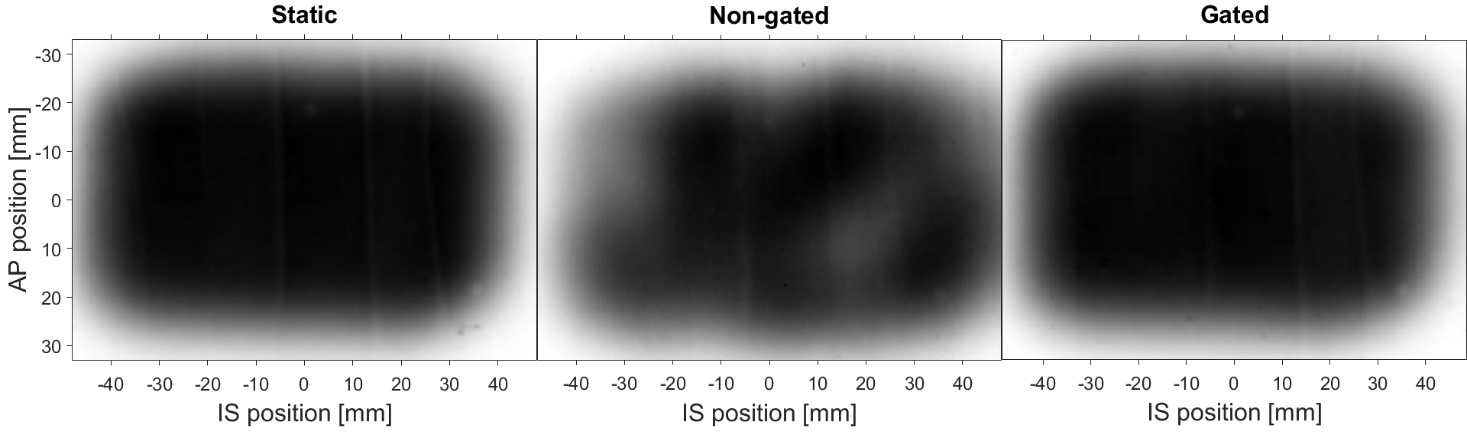


Figure S8. Comparison of blackening resulting of the moving EBT3 films after dose delivery in three modes: static (left), non-gated (middle) and gated (right). Note that the streaks (white arrows) shown in the three images are artefacts from the phantom, and not related to the irradiations. Abbreviations: AP = anteroposterior; IS = inferior-superior.
